# Cost-Effectiveness of Primary Prophylaxis of AIDS Associated Cryptococcosis in Cambodia

**DOI:** 10.1371/journal.pone.0013856

**Published:** 2010-11-09

**Authors:** Romain Micol, Ayden Tajahmady, Olivier Lortholary, Suna Balkan, Catherine Quillet, Jean-Philippe Dousset, Hak Chanroeun, Yoann Madec, Arnaud Fontanet, Yazdan Yazdanpanah

**Affiliations:** 1 Unité d'Epidémiologie des Maladies Emergentes, Institut Pasteur, Paris, France; 2 Laboratoire de Virologie, Université Paris Descartes EA 36-20, Hôpital Necker-Enfants Malades, Paris, France; 3 Mission nationale d'expertise et d'audit hospitaliers, Paris, France; 4 Institut Pasteur, Centre National de Référence Mycologie et Antifongiques, Unité de Mycologie Moléculaire, CNRS URA3012, Paris, France; 5 Université Paris Descartes, Service des Maladies Infectieuses et Tropicales, Centre d'Infectiologie Necker-Pasteur, Hôpital Necker-Enfants Malades, Paris, France; 6 Médecins Sans Frontières, Paris, France; 7 Médecins du Monde, Hopital Kosamak, Phnom Penh, Cambodia; 8 Service des Maladies Infectieuses, Hopital Calmette, Phnom Penh, Cambodia; 9 Avenir-ATIP Unit, INSERM U995, Paris, France; 10 Service Universitaire des Maladies Infectieuses et du Voyageur, CH Tourcoing, Faculté de Médecine de Lille, Lille, France; University of Cape Town, South Africa

## Abstract

**Background:**

Cryptococcal infection is a frequent cause of mortality in Cambodian HIV-infected patients with CD4+ count ≤100 cells/µl. This study assessed the cost-effectiveness of three strategies for cryptococcosis prevention in HIV-infected patients.

**Methods:**

A Markov decision tree was used to compare the following strategies at the time of HIV diagnosis: no intervention, one time systematic serum cryptococcal antigen (CRAG) screening and treatment of positive patients, and systematic primary prophylaxis with fluconazole. The trajectory of a hypothetical cohort of HIV-infected patients with CD4+ count ≤100 cells/µl initiating care was simulated over a 1-year period (cotrimoxazole initiation at enrollment; antiretroviral therapy within 3 months). Natural history and cost data (US$ 2009) were from Cambodia. Efficacy data were from international literature.

**Results:**

In a population in which 81% of patients had a CD4+ count ≤50 cells/ µl and 19% a CD4+ count between 51–100 cells/µl, the proportion alive 1 year after enrolment was 61% (cost $ 472) with no intervention, 70% (cost $ 483) with screening, and 72% (cost $ 492) with prophylaxis. After one year of follow-up, the cost-effectiveness of screening vs. no intervention was US$ 180/life year gained (LYG). The cost-effectiveness of prophylaxis vs. screening was $ 511/LYG. The cost-effectiveness of prophylaxis vs. screening was estimated at $1538/LYG if the proportion of patients with CD4+ count ≤50 cells/µl decreased by 75%.

**Conclusion:**

In a high endemic area of cryptococcosis and HIV infection, serum CRAG screening and prophylaxis are two cost effective strategies to prevent AIDS associated cryptococcosis in patients with CD4+ count ≤100 cells/µl, at a short-term horizon, screening being more cost-effective but less effective than prophylaxis. Systematic primary prophylaxis may be preferred in patients with CD4+ below 50 cells/µl while systematic serum CRAG screening for early targeted treatment may be preferred in patients with CD4+ between 51–100 cells/µl.

## Introduction

In industrialized countries, fluconazole prophylaxis is not cost-effective to prevent primary systemic fungal infections in AIDS (acquired immune deficiency syndrome) patients because of the low incidence of these infections [1], [2], [3]. Cryptococcosis is one of the most frequent and most serious opportunistic infections (OI) in AIDS patients in developing countries [4], [5], [6]. The current global burden of cryptococcal meningitis represents 957,900 cases each year resulting in 624,700 deaths during the first 3 months after infection [7]. Prevention of this opportunistic infection is a very important public health issue in developing countries [8]. In Cambodia, cryptococcosis was the leading cause of mortality among hospitalized AIDS patients in the 1990s [9], [10]. In four recent studies involving severely immunosuppressed patients with CD4+ count <100 cells/µl initiating combination antiretroviral therapy (cART), the prevalence of positive serum cryptococcal antigen (CRAG) was 20.2% (57/282) in Cambodia in 2004, 13.0% (42/336) in South Africa from 2002 to 2005, 8.8% (26/295) in Uganda during 2004–2006 period, and 12.9% (11/85) in Thailand [11], [12], [13], [14]. These studies provided evidence that systematic screening for serum CRAG was a clinically valuable strategy for early detection and targeted pre-emptive antifungal treatment. In addition, in another study performed in rural Uganda, asymptomatic positive serum CRAG was found to be an independent predictor of death during the first 12 weeks of cART in individuals with advanced HIV disease [15]. Cambodian health authorities currently recommend systematic primary prophylaxis with fluconazole in HIV-infected patients with CD4+ count <100 cells/µl (i.e. 200 mg/day orally) [16]. In 2009, the monthly cost of generic fluconazole was US$ 1.16. The cost of one time capsular cryptococcal polysaccharide detection was US$ 6.6. The aim of this study was to assess the cost-effectiveness of systematic primary prophylaxis with fluconazole (200 mg/day) or systematic serum CRAG screening and targeted treatment of positive cases compared to no intervention in HIV-infected patients with CD4+ count ≤100 cells/µl in Cambodia.

## Methods

### Analytic review

First order Monte Carlo simulation of a Markov transition model was used to project disease progression in patients with CD4+ count ≤100 cells/µl presenting to care, excluding patients with symptomatic cryptococcal meningitis. The course of the disease was modeled for one hundred thousand hypothetical HIV-infected individuals for 12 months after presentation to care. The time-horizon was therefore one year. Based on the Cambodian recommendations, it was assumed that all severely immunosuppressed patients initiated combination cART within three months after detection of HIV seropositivity. *Pneumocystis jirovecii* prophylaxis with cotrimoxazole was systematically administered to all patients. The patient trajectories with each of the following three strategies were evaluated at the time of HIV diagnosis and clinic enrollment: (1) no intervention in relation to cryptococcal prevention (comparator), (2) systematic primary prophylaxis with fluconazole 200mg/day for 6 to 12 months unless CD4+ count increased to more than 100 cells/µl, and (3) systematic serum CRAG screening and early targeted treatment of positive asymptomatic patients with fluconazole 200 mg/day after systematic lumbar puncture for 3 months. Asymptomatic serum CRAG was defined as positive serum CRAG detection with no pulmonary cryptococcosis, no symptom suggestive of meningoencephalitis (i.e. no neck stiffness, no altered mental status and no neurologic deficit), negative CRAG detection in cerebrospinal fluid, negative India ink staining in cerebrospinal fluid, and negative cryptococcal cultures in cerebrospinal fluid, blood, and urine. Patients responding to cART with a CD4+ count >100 cells/µl after 9 months of cART were dropped from intervention's cycle, as discontinuation of cryptococcosis prophylaxis during cART is considered in patients with CD4+ count >100 cells/µl [17], [18]. These patients remained however in the model until the end of the simulation without the occurrence of a new cryptococcal infection or others OI. The performance of these strategies was evaluated using incremental cost-effectiveness analysis. Incremental cost-effectiveness ratios were calculated as the additional cost (in US$) of a specific strategy compared with the cost of the next less expensive strategy, divided by the additional gain in life expectancy (per year of life gained) for this strategy. A payer perspective was adopted, i.e. based on the costs to the health system. Cost and effectiveness were not discounted since the follow-up period was less than 1 year. All costs and cost-effectiveness ratios were expressed as dollars (2009 US$) per year of life gained. A strategy ‘A’ was considered weakly dominated by another strategy ‘B’ when the incremental cost-effectiveness ratio of the strategy ‘A’ vs. the comparator (i.e. no intervention) was higher than the incremental cost effectiveness ratio of the strategy ‘B’ vs. the strategy ‘A’. A strategy ‘A’ was considered strongly dominated by a strategy ‘B’ when this latter strategy resulted in higher effectiveness and a lower overall cost than the strategy ‘A’. In addition, the performance of these strategies was estimated by determining the number needed to test and treat (i.e., with serum CRAG screening and early targeted treatment strategy) and the number needed to treat (i.e., with systematic primary prophylaxis with fluconazole) to prevent 1 death, respectively, as well as the costs associated. To estimate the cost associated to prevent 1 death, for serum CRAG screening and early targeted treatment strategy, we considered the cost of CRAG screening test and fluconazole for those tested positive. For systematic primary prophylaxis with fluconazole we considered the cost of fluconazole. We used our Markov transition model to project these costs.

### Model structure

**Figure 1:**
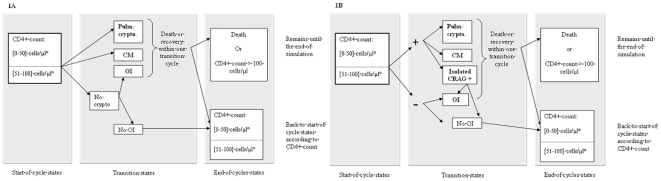
Model structure for no intervention or primary prophylaxis intervention (A), and CRAG screening intervention for treatment of positive cases (B). The probabilities of transition were stratified according to CD4+ count categories (0–50 and 51–100 cells/µl). At the end of one cycle (i.e., after 3 months), patient was back to start of cycle state within the same CD4 cell count strata or a higher CD4 cell count based on cART efficacy or was remaining alive in the model (without occurrence of new OI) because of death or CD4 cell count >100 cells/µl thanks to cART efficacy. Pulm. Crypto.: pulmonary cryptococcosis; CM: cryptococcal meningitis; OI: opportunistic infections other than cryptococcosis; +: positive serum CRAG screening; −: negative serum CRAG screening; Isolated CrAG+: Asymptomatic positive serum CRAG. Non-response to cART was included in the transition probabilities.

The model (**Figure 1**) characterized an individual patient's disease progression as a sequence of transitions from one “health state” to another, each corresponding to the patient's underlying true health. Health states were defined by current CD4+ count cells/µl and the efficacy of combination antiretroviral therapy. **Figure 1A** describes the course of patients with or without systematic prophylaxis, and **Figure 1B** with the CRAG screening strategy. At each state, patients could develop a cryptococcal meningitis, pulmonary cryptococcosis, or other opportunistic infections (any OI in patients with negative serum CRAG or with isolated positive serum CRAG). The incidence of these OIs was stratified based on CD4+ count categories (0–50 and 51–100 cells/µl). These OIs were assumed to lead to either death or full recovery. The development of an OI was also assumed not to influence the subsequent course of CD4+ count or the probability of a subsequent OI.

Patients responding to cART virologically were considered to have achieved successful immune reconstitution (CD4+ counts increasing over time). The CD4+ cell gain after three months was 47cells/µl, after 6 months was 47 cells/, and after 9 months was 27cells/µl in patients responding to cART. Patients non responding to cART were considered to decline their CD4+ count during follow-up [19], [20]. The duration of each cycle was 3 months. The non-responder status to cART was assumed to be constant during the patient's follow-up. The initial CD4+ count and non-responder status to cART of each patient were randomly selected from given distributions. The sequence of clinical trajectories for a given patient until death or end of simulation was fully determined by a set of estimated probabilities.

### Baseline characteristics and transition probabilities (Table 1)

**Table 1:** Estimates of the probabilities of events.

Variable probability	CD4+ cells/µl %		Reference
	[0–50]	[51–100]	
**CD4+ distribution in patients attending for the first time the cART program**	81	19	[11]
**Probability of cART immunological efficacy after 1 year on cART**	86.0	86.0	MSF database, [19]
**Probability CD4+ cell increase above 100 cells/µ after 6 months of successful cART**	46.0	46.0	[22]
**Asymptomatic positive serum CRAG at screening**	14.3	4.3	[11]
**Probability of CM**			MSFdatabase, [11]
Without fluconazole prophylaxis	15.4	9.1	
With fluconazole primary prophylaxis	1.0	1.0	
With fluconazole secondary prophylaxis	15.0	10.0	
With negative serum CRAG without fluconazole primary prophylaxis	0.6	0.0	
With positive serum CRAG without fluconazole primary prophylaxis	48.3	0.0	
**Probability of pulmonary cryptococcosis**			MSF database, [11]
Without fluconazole prophylaxis	3.1	1.8	
With fluconazole primary prophylaxis	0.1	0.1	
With fluconazole secondary prophylaxis	10.0	5.0	
With negative serum CRAG without fluconazole primary prophylaxis	0.000	0.000	
With positive serum CRAG without fluconazole primary prophylaxis	39.0	69.8	
**Probability of OI other than cryptococcal infection**			MSF database
For patients with negative serum CRAG	0.540	0.395	
For patients with isolated positive serum CRAG	0.540	0.395	
**Probability of death**			MSF database, [11]
In patients with CM	0.53	0.53	
In patients with pulmonary cryptococcosis	0.200	0.200	
In patients with another OI	0.200	0.200	

CM, cryptococcal meningitis; OI, opportunistic infections; cART, combination antiretroviral therapy; CRAG, cryptococcal antigen.

CD4+ distributions in HIV-infected patients seen for the first time were from two highly active antiretroviral therapy access program in Phnom Penh (Prea Bat Norodom Sihanouk Hospital - program supported by Médecins Sans Frontières, and Kosamak Hospital – program supported by Médecins Du Monde) [11]: 81% of patients had a CD4+ count ≤50 cells/ µl and 19% a CD4 cell count between 51–100 cells/µl. The probability of positive serum CRAG and mortality due to cryptococcal infection were from Micol *et al.* study in antiretroviral therapy access programs in Phnom Penh [11]. This latter study used the Latex Agglutination System (CALAS; Meridian Bioscience Europe, Nice, France). The sensitivity and specificity of the test (used in a laboratory) are 99% and 97%, respectively [21]. All patients' data routinely entered in Fuchia v1.5 (Epicentre – MSF) from the Médecins Sans Frontières Cambodian cohort in 2004–2005 were used to calculate the risk of other OIs occurrence by CD4 cell count strata [22]. Transition probabilities were stratified on two categories of CD4+ count (≤50 cells/µl versus 51–100 cells/µl) and were determined for three months (probability of cryptococcal meningitis or pulmonary cryptococcosis with or without fluconazole prophylaxis, probability of others OI, probability of death). We used data from another study performed by our group in Cambodia to estimate the CD4+ count increase in patients on efficacious cART (i.e. to estimate the time that the patients remains below 100 CD4+ cells/µl) [22]. In the latter study, we showed that in patients with a median (Q1–Q3) of CD4+ count at baseline at 20 cells/µl (6–78), the CD4+ count increased after cART initiation to reach median levels of 130 cells cells/µl (IQR, 87–189) at 6 months and 189 cells/µl (IQR, 135–250) at 12 months. In other studies, ART efficacy was estimated at 86% after one year on cART. In patients responding to cART the CD4+ cell gain was 47cells/µl after three months, 94 cells//µl after 6 months, and 121 cells/µl after 9 months. Patients non responding to cART were considered to decline their CD4+ count by 18.8 CD4+ cells/µl every three months [19], [20]. More details about the model are available in the **technical appendix S1**.

### Costs (Table 2)

**Table 2:** Estimates of the costs.

Costs in 2009 US$/3 months (MSF and MDM databases)	
Cotrimoxazole *Pneumocystis jirovecii* prophylaxis	0.89
Fluconazole prophylaxis	3.47
cART	91.5
CM/Pulmonary cryptococcosis treatment and care	187.9
Other opportunistic infections treatment and care	187.9
CRAG screening test (cost per test)	6.63

cART, combination antiretroviral therapy; CM, cryptococcal meningitis;

CRAG, cryptococcal antigen.

Costs included direct medical costs were expressed in 2009 US$. Drugs costs were from MSF cART access programs. Cotrimoxazole *Pneumocystis jirovecii* prophylaxis cost was 0.30 $/month, fluconazole prophylaxis cost 1.16 $/month, and cART treatment 30.5 $/month. Test costs and in particular CRAG screening test (6.6 $) costs were from the manufacturer (CALAS; Meridian Bioscience Europe, Nice, France) and did include the time of human resource to perform the test. To estimate costs related to cryptococosis disease and other opportunistic disease treatment and care, we considered inpatients and outpatients stays, treatment and test costs. Inpatient costs were from Médecins Sans Frontières, outpatients costs were from Médecins Du Monde. Cryptococcal meningitis (i.e.; treated with amphotericin B for 2 weeks and fluconazole 400mg/day for 8 to 10 weeks) and others OI treatment was estimated at 62.6 $/month. Patients with pulmonary cryptococcosis (mainly a severe pneumonia) received the same treatment as the patients with cryptococcal meningitis [17].

### Sensitivity analysis

One-way sensitivity analyses were performed by varying key parameters (proportions of patients with CD4+ count ≤50 cells/µl, cost of fluconazole prophylaxis, cost of CRAG screening, and mortality rate due to cryptococcal meningitis) over a wide range of reasonable values to evaluate the impact of data uncertainties and to determine the robustness of the overall conclusions.

Analyses were performed with TreeAge ProTM 2006 (Williamstown, MA, USA). This study was approved by the National Ethics Committee for Health Research of the Cambodia Ministry of Health.

## Results

### Baseline analysis

After one year of follow-up, primary prophylaxis by fluconazole or systematic CRAG screening and treatment of positive cases were more effective (probability of being alive at twelve months = 72% and 70% respectively) and more expensive (twelve months cost = 492 $ and 483 $ respectively) than the “no intervention” strategy (61%, and 472 US$) (**Table 3**). Although the “prophylaxis strategy” was more expensive than the “screening strategy” ($ 492 vs. $ 483 over a period of twelve months), it was also more effective (probability of being alive at twelve months = 72% vs. 70%). The cost-effectiveness of “screening strategy” ( = 180 $/ life year gained [LYG] vs. no intervention) was lower than the cost-effectiveness of primary prophylaxis (511 $/LYG compared to screening) (**Table 3**). Compared to “no intervention” strategy, to prevent 1 death, 10.8 persons needed to undergo CRAG screening and treatment of positive cases, and 8.9 persons needed to be treated by fluconazole prophylaxis, respectively. On the basis of the current CRAG test ($6.6/test) and fluconazole prophylaxis cost ($3.47/3 months) this translates into $77 to save 1 life with serum CRAG screening and early targeted treatment strategy and $102 to save 1 life with fluconazole prophylaxis strategy.

**Table 3:** Cost-Effectiveness of systematic fluconazole primary prophylaxis and serum cryptococcal antigen screening.

Strategy	Cost ($)	Effectiveness* (in year)	1 year survival (%)	Cost per LYG ($/LYG)	ICER ($ incr./LYG)
No intervention	472	0.741	61	636	Reference
CRAG screening+targeted therapy	483	0.803	70	601	180.0**
Fluconazole prophylaxis	492	0.822	72	599	511.0***

$: costs in 2009 US$, LYG, life year gained; CE, cost-effectiveness; ICER, Incremental cost-effectiveness ratio.

*Effectiveness = mean life expectancy one year after HIV diagnosis and clinic enrollment.

**ICER of CRAG screening vs. no intervention.

***ICER of prophylaxis vs. CRAG screening.

### Sensitivity analysis

One-way sensitivity analysis was initially performed on the proportion of patients with CD4+ count ≤50 cells/µl attending for the first time the cART access program (**Table 4**). Results were sensitive to variations in the proportion of patients with a CD4+ count ≤50 cells/µl at enrollment. If the proportion of patients attending the HIV clinic for the first time with CD4+ count ≤50 cells/µl increased by 25% to 97.3% of the clinic population (vs. 81% in the basecase analysis), the incremental cost-effectiveness ratio of both interventional strategies decreased and especially the incremental cost-effectiveness ratio of prophylaxis vs. screening decreased to 478 $/LYG. If this proportion decreased by 50% to 40.5%, the incremental cost-effectiveness ratio of prophylaxis vs. screening increased to 733 $/LYG. Finally if this proportion decreased by 75% to 20.3%, the incremental cost-effectiveness ratio of prophylaxis vs. screening increased to 1538 $/LYG. Reducing the cost of fluconazole (from 3.47 $ to 1.74 for three months) reduced fluconazole prophylaxis cost-effectiveness ratio (from 511 to 256 $/LYG respectively) (**Table 5**). When the cost of fluconazole is free (i.e. cost of fluconazole = 0 $) such as in the Pfizer Diflucan Partnership Program [23], screening strategy is weakly dominated (i.e. screening presented a higher incremental cost-effectiveness ratio than prophylaxis that is more effective) and the incremental cost-effectiveness ratio of prophylaxis vs. no intervention decreased to 141 $/LYG. Reducing the CRAG screening test cost (from 6.6$ to 3.3, or 1.7 $) decreased the incremental cost-effectiveness ratio of screening from 180, to 137, and 116 $/LYG respectively. A reduction of the mortality rate due to cryptococcal meningitis also did not inverse the results but favored the incremental cost-effectiveness ratio of screening.

**Table 4:** Sensitivity analyses: impact on the results of varying the proportion of patients with CD4+ count ≤50 cells/µl.

	Cost (2009 $)	Effectiveness* in years	Incremental CE ratio ($/LYG)**
**Increasing the proportion of patients with CD4+ count ≤50 cells/µl by 20% (i.e. 0.9732)**			
No intervention	485	0.727	
Serum CRAG screening	495	0.788	170
Fluconazole prophylaxis	506	0.811	478
**Reducing the proportion of patients with CD4+ count ≤50 cells/µl by 25% (i.e. 60.75%)** ***			
No intervention	456	0.761	
Serum CRAG screening	468	0.824	198
Fluconazole prophylaxis	474	0.835	550
**Reducing the proportion of patients with CD4+ count ≤50 cells/µl by 50% (i.e. 40.5%)**			
No intervention	439	0.783	
Serum CRAG screening	450	0.838	207
Fluconazole prophylaxis	455	0.844	733
**Reducing the proportion of patients with CD4+ count ≤50 cells/µl by 75% (i.e. 20.25%)**			
No intervention	424	0.801	
Serum CRAG screening	437	0.858	214
Fluconazole prophylaxis	439	0.859	1538

$: costs in 2009 US$, CRAG, cryptococcal antigen.

*Effectiveness = mean life expectancy one year after HIV diagnosis and clinic enrollment.

**US$ per year of life gained.

***The proportion of patients attending the HIV clinic for the first time with CD4+ count ≤50 cells/µl is 81.2% as baseline. Thus the reduction of this latter proportion by 25% decreased this proportion to 60.75%.

**Table 5:** Sensitivity analyses: impact on the results of varying the cost of fluconazole, the cost of CRAG screening test, and the cryptococcal meningitis mortality rate.

	Cost (2009 $)	Effectiveness* in years	Incremental CE ratio ($/LYG)**
**Reducing the cost of fluconazole by 50% (i.e. $ 1.74)**			
No intervention	470	0.741	
Serum CRAG screening	482	0.803	190
Fluconazole prophylaxis	487	0.821	256
**Reducing the cost of fluconazole by 100% (i.e. $ 0.0)**			
No intervention	469	0.741	
Serum CRAG screening	480	0.803	Weakly dominated
Fluconazole prophylaxis	480	0.821	141.0 (vs. no intervention)
**Reducing the cost of CRAG screening test by 50% (i.e. $ 3.315)**			
No intervention	472	0.741	
Serum CRAG screening	480	0.803	137
Fluconazole prophylaxis	492	0.821	678
**Reducing the cost of CRAG screening test by 75% (i.e. $ 1.6575)**			
No intervention	472	0.741	
Serum CRAG screening	479	0.803	116
Fluconazole prophylaxis	492	0.821	750
**Reducing the cryptococcal meningitis mortality rate by 25% (i.e. 39.75%)**			
No intervention	487	0.7683	
Serum CRAG screening	489	0.8111	44
Fluconazole prophylaxis	495	0.8250	446

$: costs in 2009 US$; CRAG, cryptococcal antigen;

*Effectiveness = mean life expectancy one year after HIV diagnosis and clinic enrollment;

**US$ per year of life gained.

## Discussion

This is the first study assessing the cost-effectiveness of three alternative prevention strategies of cryptococcal infection in HIV-infected patients. Cryptococcal opportunistic infection is highly endemic in Cambodia (estimated prevalence in HIV-infected patients with CD4+ count ≤100 cells/µl of 20.6% (58/282) in 2004 [11]) and represents more generally a significant public health burden in South East Asian AIDS patients [4], [5], [9], [24], [25]. The results of the present study suggest that, at a short term horizon of one year, the systematic CRAG screening (for targeted treatment of positive cases) in patients with CD4+ cell counts <100 cells/µl presenting to care in Cambodia, is more cost effective for cryptococcosis prevention compared to the systematic primary prophylaxis strategy. They also illustrate however that systematic primary prophylaxis strategy is more effective than systematic CRAG screening and associated with an acceptable cost-effectiveness ratio (US$ 511/LYG). Although there is no clearly defined threshold below which any health intervention can be considered to be cost-effective, the guidelines of the WHO Commission on Macroeconomics and Health can be used to establish the comparative value of alternative interventions in a given country, taking into account its ability to pay for goods and services [26]. According to these guidelines, a strategy is cost-effective if the incremental cost-effectiveness ratio is below 3× the annual per capita Gross Domestic Product (GDP) and very cost-effective if the incremental cost-effectiveness ratio is below 1× the annual per capita GDP. The Cambodian GDP was US$ 650 in 2008 (http://www.imf.org/external/pubs/ft/weo/2007/02/weodata/index.aspx). Because the incremental cost-effectiveness ratio of systematic primary prophylaxis strategy vs. CRAG screening ($ 511) is less than the Cambodian GDP, this strategy could be considered as a very cost effective strategy to prevent AIDS associated cryptococcosis in Cambodia.

The cost-effectiveness of strategies to prevent AIDS associated cryptococcosis in Cambodia estimated in our study are comparable to the cost-effectiveness of other strategies implemented in developing countries to prevent opportunistic infections occurrence in HIV-infected patients although we recognize the need for caution in directly comparing results of cost-effectiveness analysis because of differences in study design, cost components used, and the time horizon of studies. For example, the cost-effectiveness of co-trimoxazole prophylaxis for persons at early stages of HIV infection (WHO stage > or  = 2) compared to no prophylaxis has been evaluated at 150 $/LYG in Côte d'Ivoire [27] The cost-effectiveness of isoniazid preventive therapy in newly HIV-infected patients with positive tuberculin skin test has been evaluated at 102 $/QALY gained in counseling and testing centers in Kampala, Uganda [28]. In the same study, the cost-effectiveness of isoniazid preventive therapy in all newly HIV-infected patients regardless to positive tuberculin skin test has been evaluated at 106US$/QALY gained compared to the targeted testing strategy. Moreover, as far as cryptococcal infection prevention in HIV-infected patients is considered a recent study in Uganda showed that initial CRAG screening prior to starting ART in patients with CD4+ count ≤100 cells/µl is cost-effective; the number of patients needed to test and treat with CRAG screening and fluconazole to save 1 life was estimated 15.9 and the costs at $266 [13]. Systematic primary prophylaxis strategy was not evaluated in this study.

In this Cambodian analysis we found that a decrease in the proportion of patients with CD4+ count ≤50 cells/µl significantly impacted the incremental cost-effectiveness ratio of screening and prophylaxis strategies. When this latter proportion is about 20.3%, the prophylaxis strategy is a less cost effective strategy (the incremental cost-effectiveness ratio of prophylaxis vs. screening is around 1500 $/LYG). In contrast when this proportion is increased, the prophylaxis strategy becomes more cost-effective. From these data, one may hypothesize that in patients with CD4 count ≤50 cells/µl and a higher cryptococcosis incidence, the prophylaxis strategy in addition to being a more effective strategy for cryptococcal infection prevention is probably associated with an acceptable cost-effective ratio. In contrast, in patients with CD4 cell count between 51 and 100 cells/µl, prophylaxis strategy is associated with higher cost-effectiveness ratio and screening strategy may be the preferred strategy. One should consider that in addition cryptococcosis systematic screening is clinically valuable for the diagnosis of isolated positive CRAG in serum and asymptomatic cryptococcal meningitis [11], [12], [13], [14]. In the Cambodian study, of the 59 patients with cryptococcal infection, 17 (28.8%) would not have been diagnosed on the day of consultation without the agglutination test performed on serum. These patients received appropriate treatment for asymptomatic isolated positive CRAG or asymptomatic cryptococcal meningitis, which decreased either the risk of developing subsequent cryptococcal meningitis or the risk of mortality. In the absence of treatment these patients may further more develop cryptococcal immune reconstitution inflammatory syndrome [29] that is issue in South East Asia [30].

Results of the present study are based on transition probabilities drawn from real-life data and are applicable to the setting from which the results were derived. They present in addition several limitations and several points should be discussed before they can be used in the field of decision-making. We used a simulation model that combines input data from multiple sources and relies on several assumptions, although most of input data were from Cambodia. We measured resource use and effects of evaluated strategies over a 1-year-period after the time of HIV diagnosis and clinic enrollment (i.e.; the time horizon of the study one year). However, thanks to cART, HIV disease has now become a long-term chronic disease associated with a life expectancy increasing over time [31]. Long-term cost-effectiveness of cryptococcal infection prevention strategies may be different than results that we have reported in this manuscript. Nevertheless, first we believe that evaluating the cost-effectiveness of cryptococcal infection prevention in the short-term is by itself a valuable information for clinicians and policy makers. Moreover, because morbidities and mortality related to cryptococcal infections primarily occur during the first year following HIV diagnosis, estimating consequences of prevention strategies initiated at HIV care over a 1-year-period allow us to capture important effects related to these strategies. In this analysis, we were not able to estimate the incidence of each specific OI because of lack of available data and thus estimated the incidence of OIs overall. Adverse effects of drugs were not taken into account, and could be an argument in favor of the screening strategy. The exposure of cryptococcosis-free subjects to unnecessary treatment could lead to unnecessary adverse effects including ecological impact and costs. Indeed the primary prophylaxis for cryptococcal infection impact the occurrence of fluconazole resistance on Candida species [32]. Furthermore, fluconazole might have an ecological impact with the emergence of less susceptible Cryptococcus and Candida isolates. This has been well described by Bicanic *et al.* among symptomatic relapse of HIV-associated cryptococcal meningitis after initial fluconazole monotherapy [33]. Treatment adherence was assumed to be perfect, which could affect the results of both strategies, as incomplete adherence could affect the efficacy of treatment. Finally, disability weighting associated with years of life lived within categories defined in our model was not available. As such, we expressed our results in cost per year of life gained.

This study suggests that systematic serum CRAG screening and systematic primary prophylaxis strategies in patients with CD4+ count ≤100 cells/µl are very cost-effective strategies for the management of opportunistic cryptococcosis in newly diagnosed HIV-infected patients in Cambodia in 2009, although systematic primary prophylaxis was found to be more effective. Cost-effectiveness ratios associated with these strategies are in line with the cost-effectiveness of other strategies already implemented to prevent OI occurrence in HIV-infected patients in developing countries. Based on the results of this study one may consider that at the enrolment of patients in antiretroviral access program in Cambodia, patients should receive systematic fluconazole prophylaxis when CD4+ count is below 50 cells/µl and rather a systematic serum CRAG screening (for targeted treatment of positive cases) may be considered when CD4+ count is between 51–100 cells/µl.

## Supporting Information

Technical Appendix S1 (0.05 MB DOC)
