# Supplementary material for: Cost-Effectiveness of Primary Prophylaxis of AIDS Associated Cryptococcosis in Cambodia
Source: PLoS One. 2010 Nov 9;5(11):e13856. doi: 10.1371/journal.pone.0013856 (PMC2976692; doi:10.1371/journal.pone.0013856)
Supplement: Technical Appendix S1 — (0.05 MB DOC) [file pone.0013856.s001.doc]

**Technical Appendix S1**

**Introduction**

The cost effectiveness analysis described in the main manuscript is built upon three strategies concerning the prevention of AIDS associated cryptococcosis in patients with CD4+ count ≤ 100 cells/µl and without clinical signs of meningoencephalitis: (1) a no intervention strategy, which simulates the natural history of AIDS patients; (2) a systematic prophylaxis strategy, which simulates the natural history of AIDS patients initiating fluconazole 200mg/day at the time of HIV diagnosis, and (3) a serum cryptococcal antigen systematic screening which simulated the natural history of AIDS patients according to the result of the test (positive or negative) and the treatment of the cryptococcal infection. We have measured consequences of interventions in terms of effectiveness and costs and expressed our results as costs per years of life saved. (M. F. Drummond, B. O'Brien, G. L. Stoddart, and G. W. Torrance. Methods for the economic evaluation of health care programs: Oxford, Oxford University Press. 1997

M. R. Gold, J. E. Siegel, L. B. Russell, and M. C. Weinstein, eds., Cost-effectiveness in health and medicine: New York, Oxford University Press. 1996)

In this Technical Appendix, we provide greater detail on the mechanics of these strategies.

**Assumptions for the three strategies**

We used a Markov transition model to project disease progression in patients with CD4+ count ≤ 100 cells/µl presenting to care. The structure characterizes the natural history of illness in each individual patient as a sequence of trimonthly transitions from one “health state” to another and a set of estimated probabilities determine the sequence of movements between health states. Each patient’s clinical course is followed from the time of entry for 12 months. A running tally is maintained of all clinical events, the length of time spent in each health state, and the costs associated with each health state. The model estimates overall performance measures such as mean life expectancy at 12 months, proportion of patients alive at 12 months, and medical costs.

Health states predict clinical prognosis, including immune system improve or deterioration upon combination antiretroviral therapy efficacy, development of cryptococcal infection, other opportunistic infections, and mortality. The health states did not include toxic reactions to medications (cART, fluconazole and co-trimoxazole), and the potential impact of fluconazole on incidence of *Candida albicans* resistance strains.

Cryptococcal infection or other opportunistic diseases incidence have been stratified by the current CD4+ cell count (51–100 cells/µl; <50 cells/µl).

We have used variables to recall each individual’s trajectory through the process. At the start of each three months cycle, the module records the patient’s CD4+ cell count, and current therapies to determine the probabilities that indicate movement to a new state in the subsequent three months.

**T lymphocytes CD4+ distribution**

We used a categorical distribution for the CD4 cell count. At the time of HIV diagnosis 81.2% of the patients presented a CD4+ count ≤ 50 cells/µl and 18.8% a CD4+ count between [51-100]. [1]

*CD4+ proportion and distribution*

___________________________________________________

[0-50] cells/µl = 81.2% of which: [0-10] 16.24%

[11-20] 16.24%

[21-30] 16.24%

[31-40] 16.24%

[41-50] 16.24%

[51-100] cells/µl =18.8% of which: [51-60] 3.76%

[61-70] 3.76%

[71-80] 3.76%

[81-90] 3.76%

[91-100] 3.76%

___________________________________________________

**No intervention or Prophylaxis Strategy:**

At the time of HIV diagnosis patients was considered not starting (no intervention) or starting (prophylaxis strategy) fluconazole 200 mg/day orally. This prophylaxis decreased the trimonthly incidence of cryptococcal meningitis from 15.4% to 1.0% in patients with CD4+ count ≤ 50/µl, and from 9.1% to 1.0% in patients with CD4+ between [51-100] cells/µl. Then the model is the same for these two strategies. Patients entered in the model according to the distribution of the CD4+ cell count /µl (see above) with a risk of developing either cryptococcal meningitis or pulmonary cryptococcosis estimated based on their cell CD4+ cell count /µl. Following the occurrence of a cryptococcal infection patients may die or stay alive. Patients with no cryptococcal infection were exposed to the occurrence of an opportunistic infection (OI). Probabilities of death occurrence due to cryptococcal infections (53%) or other OIs (20%) were not considered to be dependant on the CD4+ cell count /µl.

We considered that the occurrence of an OI during a cycle did not impact the CD4+ count for the next cycle. Only one OI occurred by cycle. Patients on primary prophylaxis for cryptococcal infection received fluconazole 200 mg/day that was discontinued when CD4+ cell count was superior to100/µl. [2]

**CRAG Screening Strategy**

The screening module took into account the probability of serum cryptococcal antigen (CRAG) detection in HIV-infected patients without clinical signs of meningoencephalitis. It accounted for parameters such as positive serum CRAG prevalence and cost. After entering in the model according to the CD4+ cell count a serum CRAG test was performed. The patients with positive serum CRAG (14.3% in group of CD4+ count [0-50] and 4.3% in group [51-100] cells/µl) had a probability of developing cryptococcal meningitis, pulmonary cryptococcosis, or isolated positive serum CRAG. Patients with cryptococcal meningitis or pulmonary cryptococcosis presented a probability of death of 53% and 20% respectively. The efficacy of the treatment of patients with isolated positive serum CRAG was considered to be 100% [1]

Patients with isolated positive serum CRAG had a probability to develop other OIs; this probability was considered to be the same in these patients that those with negative serum CRAG.

**Combined antiretroviral therapy efficacy and the CD4+ count increase under cART [3,4]**

We considered the initiation of cART within the three first months after attending the HIV-clinic for the first time. The probability of non response after 1 year on cART was 14%. Patients were considered either responders or non-responders to cART for the whole simulation period.

In patients responding to cART, the cumulated CD4+ cell gain after three months (i.e. one cycle) was estimated to be 47cells/µl, after 6 months 94 cells/, and after 9 months 121 cells/µl.

In non responders, CD4+ cells/µl decline over three months was estimated at 18.8 cells/µl.

**References**

1. Micol R, Lortholary O, Sar B, Laureillard D, Ngeth C, et al. (2007) Prevalence, determinants of positivity, and clinical utility of cryptococcal antigenemia in Cambodian HIV-infected patients. J Acquir Immune Defic Syndr 45: 555-559.

2. Vibhagool A, Sungkanuparph S, Mootsikapun P, Chetchotisakd P, Tansuphaswaswadikul S, et al. (2003) Discontinuation of secondary prophylaxis for cryptococcal meningitis in human immunodeficiency virus-infected patients treated with highly active antiretroviral therapy: a prospective, multicenter, randomized study. Clin Infect Dis 36: 1329-1331.

3. Goldie SJ, Yazdanpanah Y, Losina E, Weinstein MC, Anglaret X, et al. (2006) Cost-effectiveness of HIV treatment in resource-poor settings--the case of Cote d'Ivoire. N Engl J Med 355: 1141-1153.

4. Madec Y, Laureillard D, Pinoges L, Fernandez M, Prak N, et al. (2007) Response to highly active antiretroviral therapy among severely immuno-compromised HIV-infected patients in Cambodia. Aids 21: 351-359.
